# Draft genome sequence data of *Erysipelothrix rhusiopathiae* vaccine strain VR-2

**DOI:** 10.1016/j.dib.2020.106352

**Published:** 2020-09-28

**Authors:** Svetlana Kovalchuk, Anna Babii

**Affiliations:** Federal Science Center for Animal Husbandry named after Academy Member L.K. Ernst, Moscow region, Russian Federation

**Keywords:** *Erysipelothrix rhusiopathiae*, Swine erysipelas, Attenuated vaccine, Draft genome sequence, WGS

## Abstract

Data on the draft genome sequence of *Erysipelothrix rhusiopathiae* strain *VR-2* is presented in this report. *E. rhusiopathiae* strain *VR-2* is a commercial attenuated vaccine widely used in Russia and a number European countries for immunization of pigs against swine erysipelas. The draft genome sequence of 1,704,727 bp in length included 1415 protein sequences, 50 tRNA genes and 3 rRNA genes according NCBI Prokaryotic Genomes Automatic Annotation Pipeline results. The draft genome sequence data of *E. rhusiopathiae* strain *VR-2* is available in GenBank under the accession nos. RJTK00000000.1, PRJNA504614 and SAMN10395786 for Genome, Bioproject and Biosample, respectively. The obtained sequence data may be helpful for searching genetic markers of *VR-2,* aimed to develop assays to discriminate between field isolates and this vaccine strain of *E. rhusiopathiae*.

## Specifications Table

**Table:** 

Subject	Biology
Specific subject area	Microbial genomics
Type of data	Genomic sequence, predicted genes and annotation
How data were acquired	Whole-genome sequencing using 454 pyrosequencing
Data format	Raw and analysed
Parameters for data collection	The genomic DNA was extracted from *E. rhusiopathiae* VR-2 vaccine strain with standard phenol-chloroform method and sequenced using 454 next generation sequencing system (Roche, Switzerland). Reads were assembled into 39 contigs using GS de novo Assembler version 2.7. Genome annotation was performed with NCBI PGAP and RAST servers.
Description of data collection	Extraction of genomic DNA, fragment library preparation, 454 pyrosequencing, de novo assembly and annotation procedures
Data source location	FSBSI «All-Russian Scientific Research and Technological Institute of Biological Industry» (FSBSI VNITIBP RAS, Shchelkovsky district, Moscow Region, Russia).
Data accessibility	Raw FastaQ reads and genome assembly data are available in Mendeley Data repository at https://data.mendeley.com/datasets/pvf8cj3zn8/2. The draft genome sequence data of *E. rhusiopathiae* strain *VR-2* is available in GenBank under the accession nos. RJTK00000000.1 (https://www.ncbi.nlm.nih.gov/nuccore/RJTK00000000.1/), PRJNA504614 (https://www.ncbi.nlm.nih.gov/bioproject/PRJNA504614) and SAMN10395786 (https://www.ncbi.nlm.nih.gov/biosample/?term=SAMN10395786) for Genome, Bioproject and Biosample, respectively.

## Value of the Data

• Draft genome data may be used for study in depth the genetic features of this *E. rhusiopathiae* vaccine strain.
• Draft genome data may be helpful for searching genetic markers of *E. rhusiopathiae* vaccine strain VR-2 aimed to develop genetic assays for differentiation of field isolates and this live vaccine strain.
• This genome sequence data may be used in comparative studies to better understand the genetic diversity and the genomic and molecular attributes underlying the pathogenicity of *E. rhusiopathiae* strains.

## 1 Data Description

*Erysipelothrix rhusiopathiae* is a gram-positive, non-spore-forming, rod-shaped bacterium that causes erysipeloid in humans and erysipelas in animals, including swine erysipelas [1], [2], [3]. Due to economical importance of swine erysipelas in husbandry, an epizootic of *E. rhusiopathiae* is controlled by administration of commercially available *E. rhusiopathiae* vaccines in many countries [4], [5], [6], [7], [8]. In Russia and a number of European countries *E. rhusiopathiae* live attenuated vaccine strain VR-2 is widely used for this purpose [9], [10], [11]. The use of attenuated vaccine strains is associated with risks of their reversion to virulence, caused outbreaks of swine erysipelas, therefore differentiation of *E. rhusiopathiae* vaccine strain VR-2 and field isolates is especially important for epizootical monitoring of swine erysipelas in countries applying this commercially available live attenuated vaccine for immunization of pigs against swine erysipelas. In this regard whole genome sequencing is a promising approach for searching for genetic markers of *E. rhusiopathiae* VR-2 to develop the strain-specific genetic assays for differentiation of field isolates and this live vaccine strain.

After assembling the obtained 89,701 sequence reads 39 contigs of 1,704,727 bp total length at 20-fold coverage were generated (Table 1). A 36.5% CG content of VR-2 strain well correlated with CG% of the complete genome sequences of other *E. rhusiopathiae* strains form GenBank (Table 1). VR-2 genome sequence identity with genome neighbors – *E. rhusiopathiae* strains WH13013 and Fujisawa – was 94.9695% and 94.7062%, respectively.

**Table 1:** General features of the *E. rhusiopathiae* strain VR-2 genome sequence.

Feature	Value
Size	1,704,727
Coverage	20x
GC Content	36.5%
N50	83,301
L50	7
Number of contigs	39

**Fig. 1.**
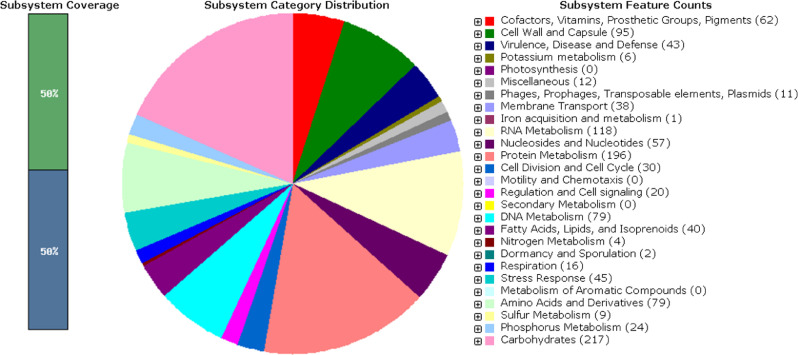
An overview of the subsystem statistics for draft genome sequence of *E. rhusiopathiae* strain VR-2 generated by Rapid Annotation System Technology (RAST).

Annotation of the assembled data with NCBI Prokaryotic Genomes Annotation Pipeline [12,13] revealed 1415 protein sequences, 50 tRNA genes and 3 rRNA genes. Annotation with the RAST server [14,15] revealed 264 subsystems. An overview of the subsystem statistics for the draft genome sequence of *E. rhusiopathiae* strain *VR-2* generated by Rapid Annotation System Technology (RAST) is shown in Fig. 1.

Raw FastaQ reads, the genome assembly data and results of the draft genome sequence annotation with RAST server are available in Mendeley Data repository at https://data.mendeley.com/datasets/pvf8cj3zn8/2.

## 2 Experimental Design, Materials and Methods

DNA sample of *E. rhusiopathiae VR-2* vaccine strain were kindly provided by FSBSI «All-Russian Scientific Research and Technological Institute of Biological Industry» (FSBSI VNITIBP RAS, Shchelkovsky district, Moscow Region, Russia). The genomic DNA was extracted and purified with standard phenol-chloroform method.

The genomic DNA was sequenced using 454 next generation sequencing system (Roche, Switzerland). In brief, 500 mg of the genomic DNA was fragmented by nebulization using Rapid Library Nebulizers (Roche, Cat. No. 05233780001) and Rapid Library Buffers (Roche, Cat. No. 05619181001). The fragmented DNA was purified on the columns from the Qiagen MiniElutePCR Purification kit (Cat. No. 28004) and ligated with DNA adaptor using Rgt/Adaptors kit (Roche Cat. No. 05619203,001) according to the manufacturer's instructions. Small DNA fragments and unligated DNA adaptor molecules were removed using Agencount AMPure XP Beads (Beckman Coulter, Cat. No. A63880). The obtained DNA library was qualified using Lonza FlashDoc (Cat. No. 57025) and FlashGel DNA Cassette (Lonza, Cat. No. 57023). The DNA fraction with predominant sizes ranging from 500 to 800 bp was quantified with Quant-it Picogreen dsDNA Assay Kit (Invitrogen, Cat. No. P7589) and QuantiFluor-ST Fluorometer (Promega, Model E6090) and then amplified by an emulsion PCR with the use of emPCR Reagents Lib-L Kit (Cat.#05996503001). The prepared DNA library was added to the emulsion PCR at a ratio of two DNA molecules per bead. After emPCR completion the emulsion was breaking with the use of Oil and Breaking Kit (Roche Cat. No. 05996511001). The beads with the amplified DNA was isolated with Bead Recovery Reagents (Roche, Cat. No. 05996490001). Sequencing of the DNA library was performed using Sequencing Buffers (Roche, Cat. No. 05996589001), Reagents and Enzymes (Roche, Cat. No. 05996562001), Packing Beads & Supplement CB (Roche, Cat. No. 05996597001) and PicoTiterPlate Kit (Roche, Cat. No. 05996619001). The beads with the amplified DNA as well as layers of packing, enzyme and PPiase beads were loaded into the wells of PicoTiterPlate according to the manufacturer's instructions and sequenced using GS Junior instrument (Roche, Switzerland). Reads were assembled into contigs using the GS de novo Assembler (version 2.7) with default parameters.

Genome annotation was performed using the NCBI Prokaryotic Genomes Automatic Annotation Pipeline [12,13] and Rapid Annotation System Technology (RAST) server [14,15]

## Author Statement

The authors contributed equally to this work.

## CRediT authorship contribution statement

**Svetlana Kovalchuk:** Conceptualization, Methodology, Formal analysis, Writing - original draft. **Anna Babii:** Investigation, Visualization, Writing - review & editing.

## Declaration of Competing Interest

The authors declare that they have no known competing financial interests or personal relationships which have, or could be perceived to have, influenced the work reported in this article.
